# Analysis of intra-particle liquid capillary spread mechanisms in high-temperature stope leaching using MRI

**DOI:** 10.1038/s41598-022-09154-4

**Published:** 2022-03-28

**Authors:** Zhenlin Xue, Deqing Gan, Youzhi Zhang, Zhiyi Liu

**Affiliations:** College of Mining Engineering, North China University of Science and Technology, 21 Bohai Road, Caofeidian Xincheng, Tangshan, 063210 Hebei China

**Keywords:** Hydrology, Engineering, Physics

## Abstract

Capillary penetration is widely existed in stope leaching, both the rate of liquid wetting ore and flow out of ore are affected by it. Stope leaching is carried out in a high-temperature environment when mining minerals with large burial depth. The mechanism of intra-particle liquid capillary penetration mechanisms at high-temperature have not been revealed. In this paper, samples with a size of Φ50 mm × 100 mm were selected for quantitative analysis. The capillary rise behaviour inside samples with different porosity were detected at 30 °C, 40 °C and 50 °C by using magnetic resonance imaging (MRI). In most cases, capillary rise height is underestimated when the outside wetting line is used as an indicator, because the rise height inside the sample is greater. The liquid capillary rise height increased slightly with the temperature, whereas the wetting surface profile remained unchanged. The capillary rise rate increased significantly with porosity, mainly due to the increase of internal effective porosity. The results help to understand the liquid penetration behaviour under high-temperature stope leaching condition, and lay a theoretical foundation for improving the liquid permeability.

## Introduction

Stope leaching technology can effectively deal with the problems faced by underground mining (e.g. high in-situ stress, high temperature and high lift length), and has broad prospects to extract valuable metals from low-grade copper, gold, silver, and uranium ores with large burial depth^1,2^. The liquid penetration in packed beds is a key factor affecting the mineral extraction^3^. The transportation of chemical reagents to the ore and that of the extracted minerals, along with temperature adjustment, are achieved through liquid penetration^4,5^. However, several permeability problems, such as uneven distribution of solution, preferential flow, and surface runoff, limit improvements in extraction efficiency^6,7^.

Studies have explained the liquid penetration behaviour in the packed beds, which is affected by multiple complex factors. Liu and Hashemzadeh^8^ reported the relationship between the irrigation rate, dripper spacing, packed beds height, initial moisture content, and the time taken for the liquid to flow out of the packed beds. The uneven moisture distributions in the steady state are more significant under higher irrigation rates and larger dripper spacings. Hydrodynamics is greatly affected by the form of liquids addition, and intermittent liquid addition significantly improves the liquid distribution uniformity^9^. Under extreme low-temperature conditions, the liquid flow is enhanced when the temperature increases, whereas the influence of irrigation rates on the liquid flow is limited^10,11^. The hydrodynamics is also affected by particle accumulation. Particle segregation leads to an uneven pore distribution, wherein the liquid flows rapidly through the large pores, resulting in a preferential flow^12–15^. Several measures, such as antiscalant addition, ore washing, classified crushing and screening, thin-layer conveying, and dumping, can effectively improve the poor permeability in industrial leaching^16,17^. The mineralogical composition of ores, such as clay, affects permeability. Swelling occurred almost instantaneously after water clay contact, which results in a decrease in porosity and permeability. The permeability decreases as the clay content increases. The aforementioned studies indicate that the ore particle size distribution, packed beds shape parameters, physical and mechanical properties of the ore, liquid addition, environmental temperature, and chemical reaction significantly affect the hydrodynamics, and the results of these studies have proved beneficial for improving industrial leaching. However, these studies all focussed on the relationship between the parameters (liquid addition, shape parameters, etc.) and macroscopic permeability.

The macroscopic permeability is determined by liquid penetration behaviour inside the packed beds. A comprehensive understanding of the liquid penetration behaviour can help understand the fundamental penetration mechanisms^18^. Liquid distribution and spread mechanisms can be revealed by non-invasive detection techniques^19^. Based on magnetic resonance imaging (MRI) technology, the inter-particle pores and liquid distribution have been identified and quantified in the saturated state. The flow velocity distribution in pores to be nearly parabolic^13^, and the formation of new rivulets significantly increases with irrigation rate^20–22^. To investigate the liquid spread in a pseudo 2-D column, ultraviolet light was used^6,23^. An extensive horizontal spread, quantitatively characterised by the lateral spread coefficient, was observed in packed beds. Based on inter-particle pore structures obtained by computed tomography (CT) or MRI, flow models have been used to simulate the flow behaviour in pores^24^. Lin et al.^25^ used a milli-CT scanner to determine the pore structure before and after copper ore bioleaching and determined the saturated flow field based on lattice Boltzmann (LB) simulations. Miao et al.^26,27^ used CT to determine the two-scale pore network in column leaching and simulated the liquid flow at the inter-particle scale based on the Navier–Stokes and Brinkman equations. Li Tao^28^ obtained the pore structure based on MRI, combined the discrete element method and lattice Boltzmann method (LBM) to conduct numerical simulations, and obtained the relationship between the maximum flow velocity and the irrigation rate. Fernando et al.^29,30^ obtained the pore structure of a pseudo 2-D packed bed using CT technology and proposed a flow model using a weighted random walk approach to ascertain the probabilistic behaviour of the gravity-dominated flow features. All these studies focussed on the inter-particle flow in the packed beds, the intra-particle slow flow behaviour (capillary penetration) in the underground temperature environment was not analysed. The inter-particle flow is a free flow under the gravity action, while the intra-particle is very slow capillary penetration.

Liquid capillary permeation, including liquid infiltration of ore and the transportation of valuable minerals out of the ore with liquid, is widely existed in the leaching process. Ilankoon^6,23^, Yin^31^, Xue^2^, and Miao^27^ concluded that capillary permeation affects the liquid spread and mineral extraction in packed beds. However, only a few reports the capillary penetration. Yin^31^ studied the influence of particles and accumulation pores on lateral capillary permeation, and Mikhailov^32^ reported on the capillary rise during column leaching. These studies focussed on the height evolution of the wetting line on the column surface and capillary water absorption. However, the intra-particle capillary rise mechanisms at high-temperature condition of stope leaching were not explored.

However, due to the irregular ore shape after crushing, the capillary penetration inside the ore is difficult to be quantified. To address the aforementioned gaps in the research, in this study, the intra-particle capillary rise in stope leaching was innovatively analysed using MRI to determine the liquid distribution non-invasively. Cylindrical rock samples^33^ are selected to reduce the influence of shape factors and to obtain quantitative results. The influence of ore porosity and high-temperature on the capillary rise was investigated, and liquid capillary rise mechanisms were analysed. The study results can be used to establish a more accurate double permeable media model of stope leaching, and facilitate a comprehensive understanding of stope leaching hydrodynamics.

## Materials and methods

### Materials

Valuable minerals are randomly distributed in ores. During the leaching process, the minerals are extracted and the pores in the samples change irreversibly. Thus, the results of capillary rise experiments using ore samples are unrepeatable. To obtain accurate repeatable experimental results, in this study, the ore materials and leaching liquid used were sandstone and distilled water, respectively. As sandstone is much more uniform than ore. Three porosity sandstones of A, B and C are selected respectively, and the corresponding porosities are 2.1%, 14.6%, and 17.3% respectively. Because the ore shapes in packed beds are irregular, the experimental samples need to be fixed in order to obtain the mechanism of capillary penetration. The sandstones were processed into standard cylindrical samples of size Φ50 mm × 100 mm, according to the relevant standards of rock experiments^34^. And dried at 105 °C for 48 h before the experiment. The initial weight was recorded.

### Experimental device

**Figure 1:**
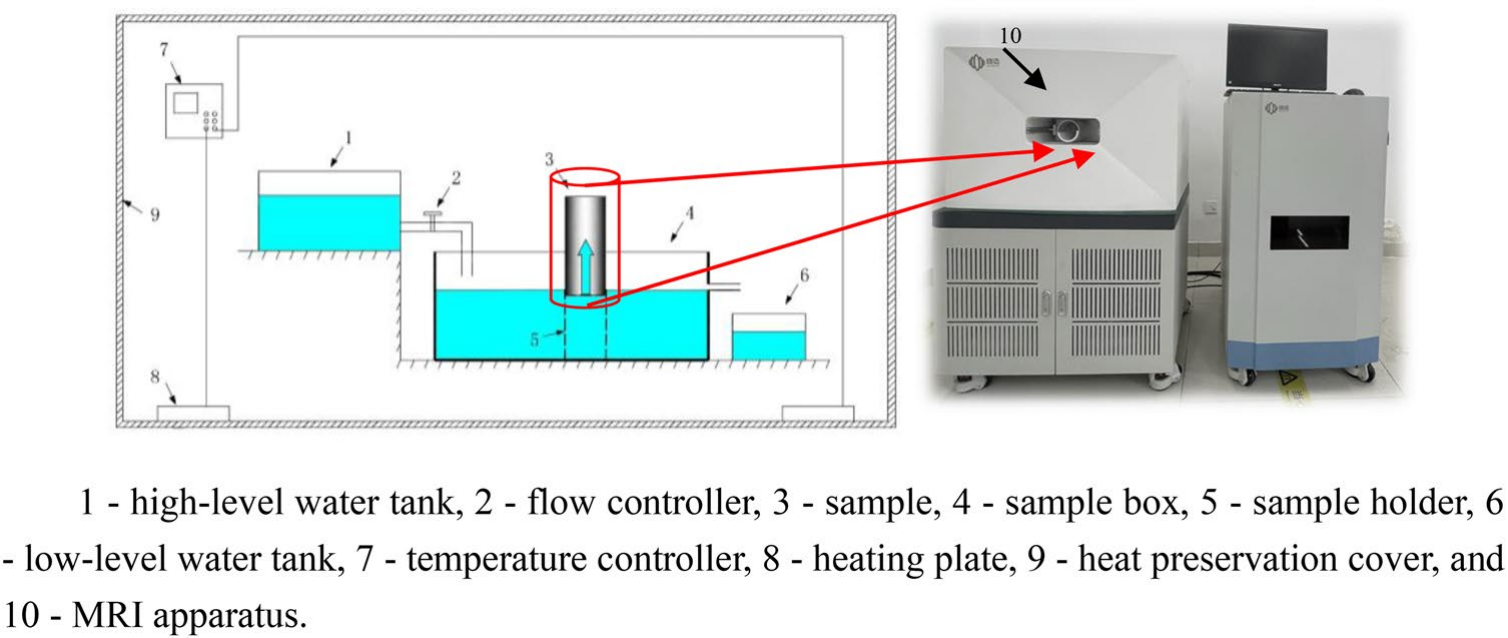
Capillary rise device.

The experimental device consisted of two parts: a capillary rise device and an MRI device. The capillary rise device was self-made and included a high-level water tank, flow controller, sample box, low-level water tank, sample holder, temperature controller, heating plate, sample, and heat preservation cover, as depicted in Fig. 1. The high-level water tank continuously injects liquid into the sample box, and the flow controller controls the flow rate. The water outlet is set on the right of the sample box to maintain a constant liquid level. The heat preservation cover, made of high-rated light transmission plexiglass, is convenient for data recording during experiments. The sample holder is designed to be 1.0 cm lower than the liquid level to reduce the influence of minor liquid fluctuations on the capillary rise. The MRI device is a 0.5-T low-field device manufactured by Numai Technology, China. The magnetic field strength is 0.5 ± 0.08 T, the main frequency of the instrument is 21.3 MHz, and the probe coil diameter is 60 mm.

### Basic principles of nuclear magnetic resonance (NMR) experiments

The basic principles of T_2_ relaxation characteristics for measuring the liquid distribution are as follows. In a uniform magnetic field, the T_2_ transverse relaxation of fluid in rock pores follows three mechanisms, namely, free relaxation T_2B_, surface relaxation T_2S_, and diffusion relaxation T_2D_. These three relaxations have the following relationship:

1 $$ \frac{1}{{T_{2} }} = \frac{1}{{T_{2B} }} + \frac{1}{{T_{2S} }} + \frac{1}{{T_{2D} }} $$

Under the experimental conditions, the free and diffusion relaxations are significantly lower than the surface relaxation. Therefore, considering T_2_ to be equal to T_2S_,

2 $$ \frac{1}{{T_{2} }} = \frac{1}{{T_{2S} }} = \rho_{2} \left( \frac{S}{V} \right)_{P} = \frac{{\rho_{2} \cdot Fs}}{r} = \frac{C}{r} $$

Here, ρ_2_ is the relaxation rate (μm/ms), S/V is the ratio between the liquid surface area and volume in the pore, F_S_ is the shape factor (dimensionless), and r is the pore radius in μm.

The internal pores of sample are mainly primary pores, which are mainly the pore between primary mineral crystal particles. The three samples are all from the same region and have the same pore type. Therefore, Fs of different samples is considered to be the same. The relaxation rate ρ_2_ and the pore shape factor F_S_ are regarded as constants, and thus, the coefficient C is a fixed value. The lateral relaxation time is proportional to the liquid radius in the pore. Therefore, the signal strengths of different lateral relaxation times can characterise the intra-particle liquid distribution.

The basic principles of MRI are based on the different attenuation speeds of energy released inside a sample. The position and type of nuclei in the sample are determined by applying a gradient magnetic field to detect the emitted electromagnetic waves and obtain internal images.

### Experimental procedures

To reproduce the results, three samples with the same parameters were selected for each experiment set. Sufficient distilled water was added to the high-level water tank and the sample box. After the samples were placed inside the heat preservation cover, the temperature control device was turned on. When the temperature in the heat preservation cover reached the pre-set values (30 °C, 40 °C, and 50 °C) and remained stable for 10 min, the samples were placed on the holder simultaneously. After 10 min, the samples were taken out, and their weights and heights of the wetting lines were recorded. Subsequently, the samples were placed inside the MRI equipment to start the next set of experiments. A 75-mm probe was used based on the core diameter, and the marked section was used as the probe reference plane. The free induction decay and Carr–Purcell–Meiboom–Gill^35^ sequences were used in the experiment. The main parameters settings in the experiment for the T_2_ relaxation were SW = 200, RFD = 0.002, and RG1 = 20, and those in the imaging experiment were TE = 5.885, TR = 700, averages = 16, GA1 = 34.5, and GA4 = 13.5, the slice thickness was 50 mm, which mean the entire sample was detected as a slice. The samples were dried for 48 h following the experiment. The capillary rise and NMR experiments were repeated, with the capillary rise times set to 20 min, 40 min, 80 min, and 160 min. Each experiment was repeated until reproducible results were obtained.

## Results and discussion

### Liquid capillary rise characteristics

**Figure 2:**
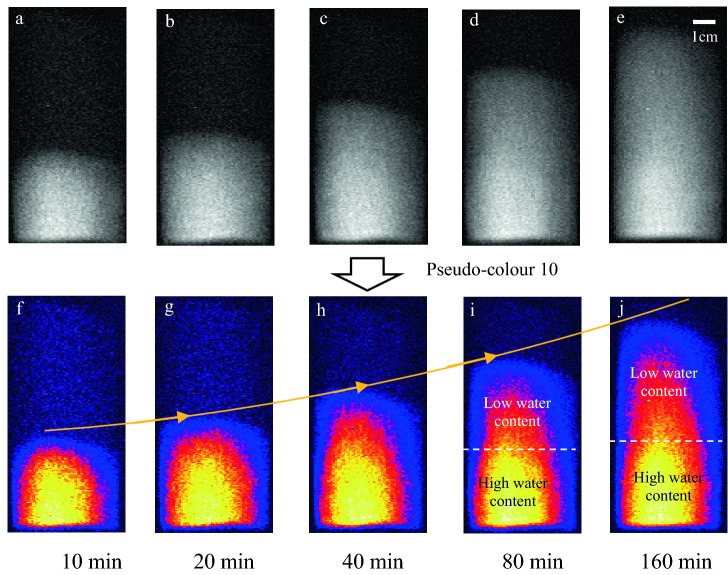
Liquid capillary rise characteristics with time.

The MRI experimental results are grayscale images. To facilitate comparison and analysis, the grayscale images are processed with false colour using image processing technology. The intra-particle liquid capillary rise characteristics of sample C at 50 °C are presented in Fig. 2. In the radial direction, the liquid is unevenly distributed, with a larger amount present in the centre and a lower amount at the edges. This is because the cylindrical sample is viewed as one slice in the vertical direction during MRI. The liquid moves upwards along the micropores owing to the surface tension acting at the liquid–solid interface, and the internal wetting surface height of the sample increases with time. The microscopic process is as follows: when the liquid penetrates into the micropores, its concave surface increases the surface area. Owing to the attractive force between the pipe wall and water molecules, the surface free energy changes the liquid surface profile, raising the liquid level inside the micropores. The wetting between water and pipe wall causes subsequently liquid surface to revert to a concave shape. The aforementioned process continues, and the liquid gradually moves upwards inside micropores^36^.

Figure 2 shows that the liquid rises to a higher position at 80 min and 160 min and the water content in the upper part drops significantly. The liquid spreading along the micropores under capillary action and effective connection between pores are prerequisites for the liquid capillary rise. With the height increase, the capillary rise path increases and the capillary rise rate decreases. From this perspective, the path for the liquid to infiltrate the ore through capillary permeation is long in large ores, and a reasonable ore size enables more efficient stope leaching.

**Figure 3:**
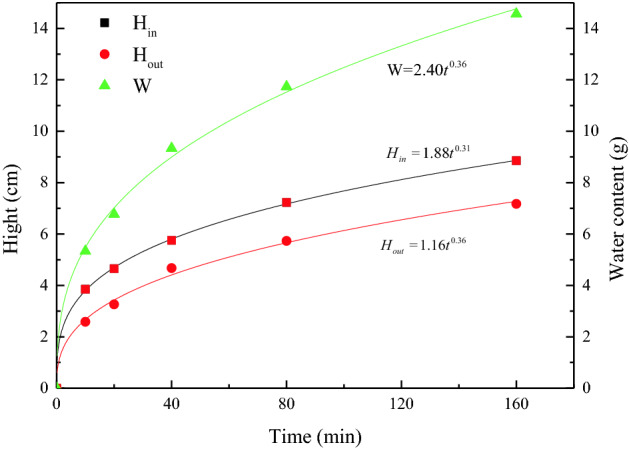
Evolution of capillary rise parameters with time: H_in_: height of the internal wetting surface, H_out_: height of the external wetting line, and W—water content.

Figure 3 presents the relationship between the liquid rise parameters and time, to quantify the characteristics of capillary rise, and the error bars are small and hide in the symbols. The water content (W) and the heights of the internal wetting surface (H_in_) and external wetting line (H_out_) increase in power functions with time. However, the increase rate gradually decreases, which is consistent with the results of Mikhailov^32^ and Yin^31^. When the sample is in contact with the liquid, the bottom is immersed in water, and the internal space is quickly filled by the liquid and tends to become saturated. This process includes wetting and capillary actions, and the wetting action rate is much greater than that of capillary action. After passing through the submerged part, the liquid rises mainly by capillary action. Based on the balance between gravity and capillary suction force, the equation for calculating the maximum capillary rise height is as follows^37^:

3 $$ h_{c} = \frac{4\sigma \cos \alpha }{{d\gamma_{w} }} $$

where h_c_ is the capillary rising height (m), $\sigma$ is the surface tension between water and air (N/m), $\alpha$ is the wetting angle (°), d is the capillary diameter (m), and $\gamma_{w}$ is the water bulk density (KN·m^-3^).

The wetting angles of three samples are considered to be similar. Equation (3) shows that the main factors affecting the capillary rise are the capillary diameter and gravity. The capillary diameter does not change when the liquid capillary rises. At the beginning of the capillary rise, the capillary suction is much larger than gravity as the quantity of water in the capillary is less and the rising rate is high. However, as the capillary water rises, the gravity increases, gradually reducing the rising rate. When the capillary suction and gravity reach equilibrium, the capillary water stops rising.

Although the upward trends of H_in_ and H_out_ are similar (Fig. 3), H_in_ remains greater than H_out_ throughout the process. An error exists between the traditional wetting line data and the rising height inside the sample. The error range and percentage are 1.1–1.7 cm and 23–49%, respectively. In summary, although the traditional method of measuring the wetting line reveals the capillary rise trend, the external visual measurement underestimates the capillary rise hight.

**Figure 4:**
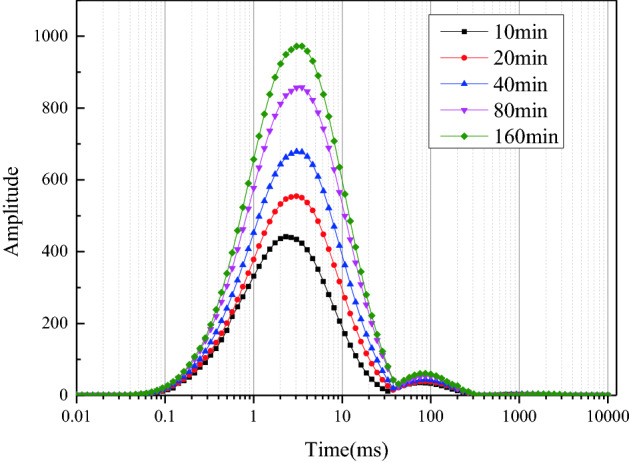
T_2_ relaxation characteristics.

Figure 4 reveals that the relaxation time of the main peak in the T_2_ curve is 2–3 ms. This implies that the liquid is mainly distributed in the micropores in the form of bound water and cannot flow^38^. The peak value increases with time, indicating that most of the liquid exists on the micropore walls or is wrapped around the surface of particles during the capillary rise process. The relaxation time of the second peak is 30–300 ms and is indicative of capillary water distributed in well-connected pores or pore water in large pores. The second peak value is low, and the peak value and maximum relaxation time increase only slightly with time; this indicates that the liquid content in the elongated pores or relatively large pores is low during the capillary rise process. These results are consistent with the MRI results, as show in Fig. 2i, j.

### Effect of porosity

**Figure 5:**
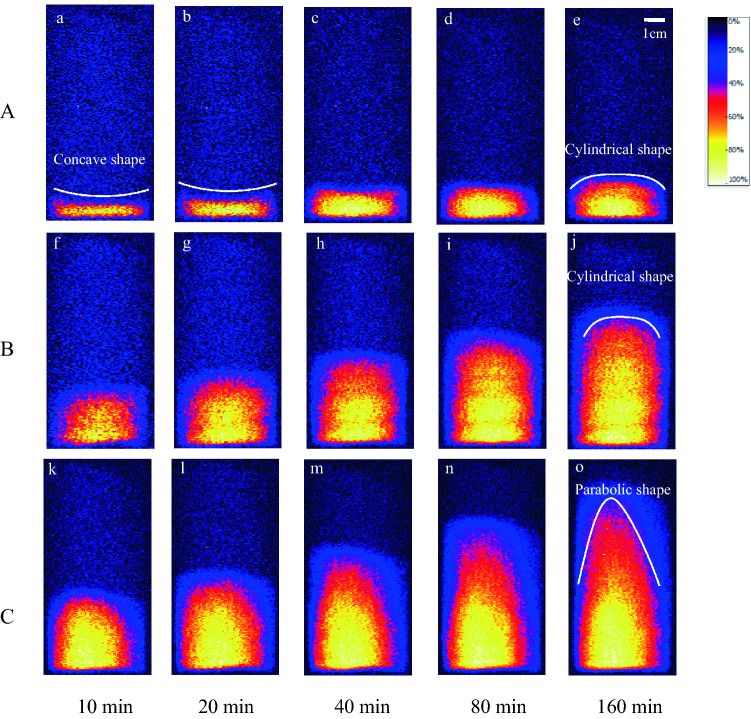
Liquid capillary rise characteristics with different porosities (50 °C).

Effective porosity, an important parameter affecting the liquid capillary penetration, is the ratio between the volumes of interconnected pores and the sample. Figure 5 shows that the liquid capillary rising height and distribution are significantly affected by porosity, and that the rising height increases substantially with porosity. The internal pores of low-porosity particles are small and largely disconnected, resulting in a low effective porosity and limiting the liquid capillary rise^39^. Conversely, the internal pores of high-porosity particles are large and have increased connectivity, resulting in a high effective porosity and significantly increasing the liquid rising height.

The liquid distribution during capillary rise changes with an increase in particle porosity. The internal wetting surface of low-porosity particles is distributed in a columnar shape, and the top wetting line is distributed nearly horizontally. In high-porosity particles, the liquid wetting surface exhibits a parabolic distribution, which is evident after 40 min owing to the increase in effective porosity. The pores in the high-porosity particles are large and evenly distributed. The sample is cylindrical, and the liquid rises faster along the centre of the sample, thus forming a parabolic shape.

The liquid distribution during the capillary rise in sample A, a low-porosity sample, is significantly different from that in the other samples. The liquid height at the edge is substantially higher than that at the centre at 10 min and 20 min. This is due to the time for the liquid to wet the sample bottom increases as porosity decreases. When the sample is placed in the liquid, the liquid slowly penetrates the interior from the edge, and the immersed sample bottom cannot be wetted completely in 20 min. The liquid simultaneously moves upwards along the micropores at the edge, resulting in a greater liquid rising height.

**Figure 6:**
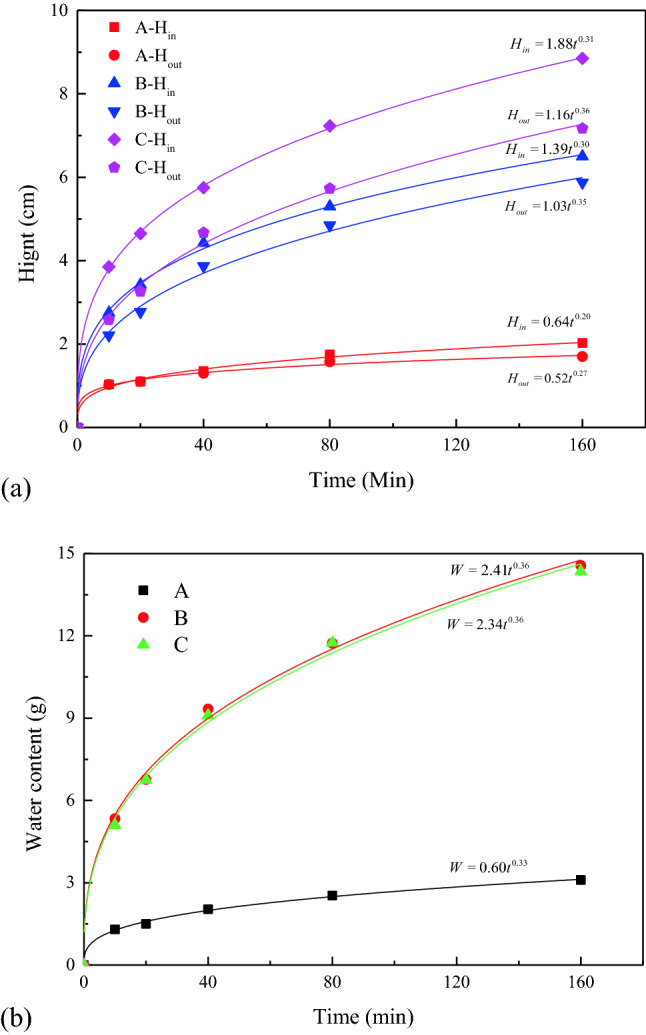
Evolution of capillary rise parameters in samples with different porosity.

In Fig. 6 the error bars are small and hide in the symbols. Figure 6a indicates that H_in_ and H_out_ of different samples increase in power functions with time. And the rising height increases with porosity, which indicates that high porosity is beneficial to the valuable mineral extraction. Figure 6a indicates that capillary rise height is underestimated when the wetting line outside the sample is used as an indicator, because the H-in of the sample is greater. And the difference value between H_in_ and H_out_ increases with porosity. Figure 6b indicates that the W of samples with different porosities increases in power functions with time, while the water absorption rate gradually decreases. Although the capillary water absorption increases with porosity, the water absorptions of samples B and C are almost identical at different time intervals; this indicates that the absorption in these samples does not increase significantly after the porosity reaches a certain level. An increase in capillary length with porosity promotes capillary water absorption, whereas an increase in capillary diameter reduces the capillary water absorption rate^40^. The combination of two factors results in capillary water absorption not increasing continuously with the porosity.

Based on Figs. 5f–o and 6b, the liquid distribution is significantly different in samples B and C with equivalent water absorption. The capillary rise height of sample C is greater, and the liquid distribution in sample B is more balanced. These findings demonstrate that traditional water absorption measurement method cannot reflect the liquid capillary rise characteristics accurately.

### Effect of temperature

**Figure 7:**
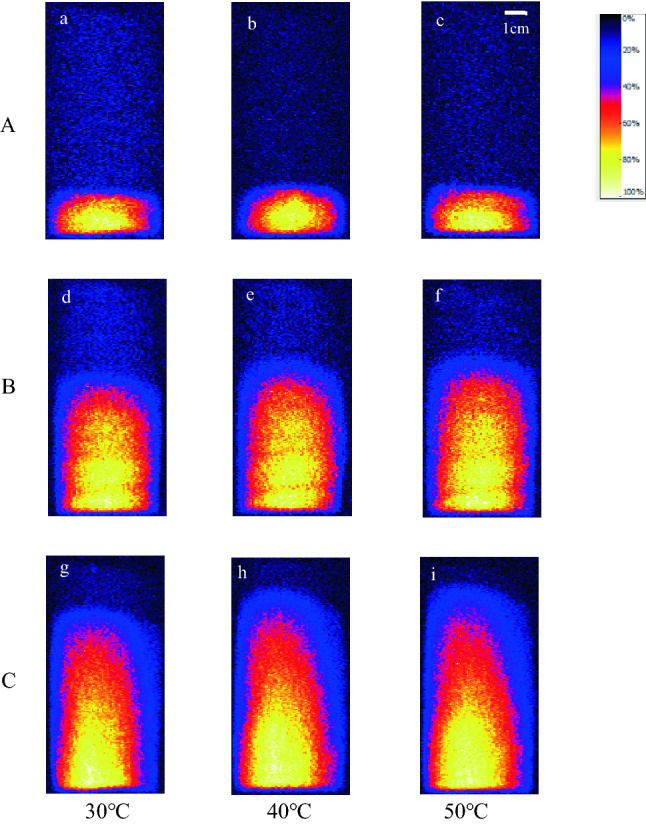
Liquid capillary rise distribution with different temperatures (160 min).

In the first 80 min, the difference of capillary rise height is very limited at different temperatures, and only the liquid distribution at 160 min is shown in Fig. 7. Figure 7 reveals that the liquid capillary rise increased slightly with the temperature. And the liquid distribution profile remained unchanged at different temperatures. The liquid exhibited a columnar distribution in samples A and B, while a parabolic distribution in sample C. The influence of temperature on the liquid capillary rise mainly included the following aspects: (a) Micropores in rocks. The temperature affects micropores only when it is within a certain range, which is generally 150–300°C^41,42^. In this study, the temperature was maintained below the threshold. The distance between mineral particles increased slightly owing to the thermal stress change caused by thermal energy, which led to a slight deformation of pores and enhanced the pore connectivity^43^. (b) Reduced liquid viscosity. When the temperature increased from 30 °C to 50 °C, the kinematic viscosity of water decreased from 8.04 × 10^–7^ to 5.56 × 10^–7^ m^2^/s, and the liquid fluidity increased, thereby slightly increasing the liquid flowing speed in the capillary. (c) Reduced water density. When the temperature increased from 30 °C to 50 °C, the water density decreased from 995.6 to 988.0 kg·m^-3^. The capillary rise height increased as the liquid density decreased, according to Eq. (3). (d) As the temperature increases, the internal energy of liquid increases, and the irregular movement of liquid molecules becomes more intense, resulting in enhanced fluid diffusion in the sample.

**Figure 8:**
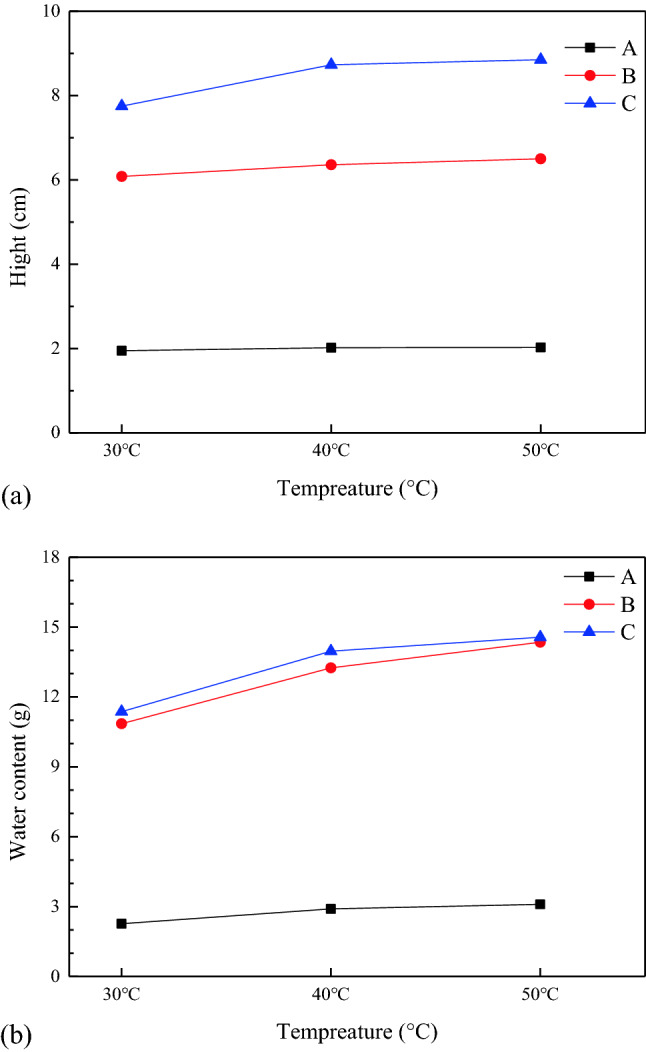
Influence of temperature on capillary rise height and water content.

In Fig. 8 the error bars are small and hide in the symbols. Figure 8 reveals that the liquid rising height and capillary water absorption slightly increase with temperature, and it is more significant in the higher-porosity samples (B, C). The influence of temperature on water absorption is greater than capillary rise height. In terms of temperature ranges, the capillary rise height and water absorption changed significantly from 30 °C to 40 °C, while the change was relatively small from 40 °C to 50 °C. When the temperature increased from 30 °C to 40 °C the kinematic viscosity decreased by 1.45 × 10^-7^m^2^/s. And the kinematic viscosity decreased by 1.03 × 10^–7^ m^2^/s with temperature increase from 40 °C to 50 °C.

To summarise, when the temperature is within the threshold, the thermal stress caused by the temperature rise is not sufficient to cause a considerable change in the shape and number of pores, and the fluidity of liquid and changes in density are extremely limited. Capillary water absorption and capillary rise height do not increase linearly with temperature. For industrial stope leaching, the temperatures are in the range below the threshold. Therefore, an increase in ambient temperature and the exothermic heat of the leaching reaction in the stope accelerate liquid penetration into ores, improving the leaching efficiency of valuable minerals.

### Coupling effect of porosity and temperature

**Figure 9:**
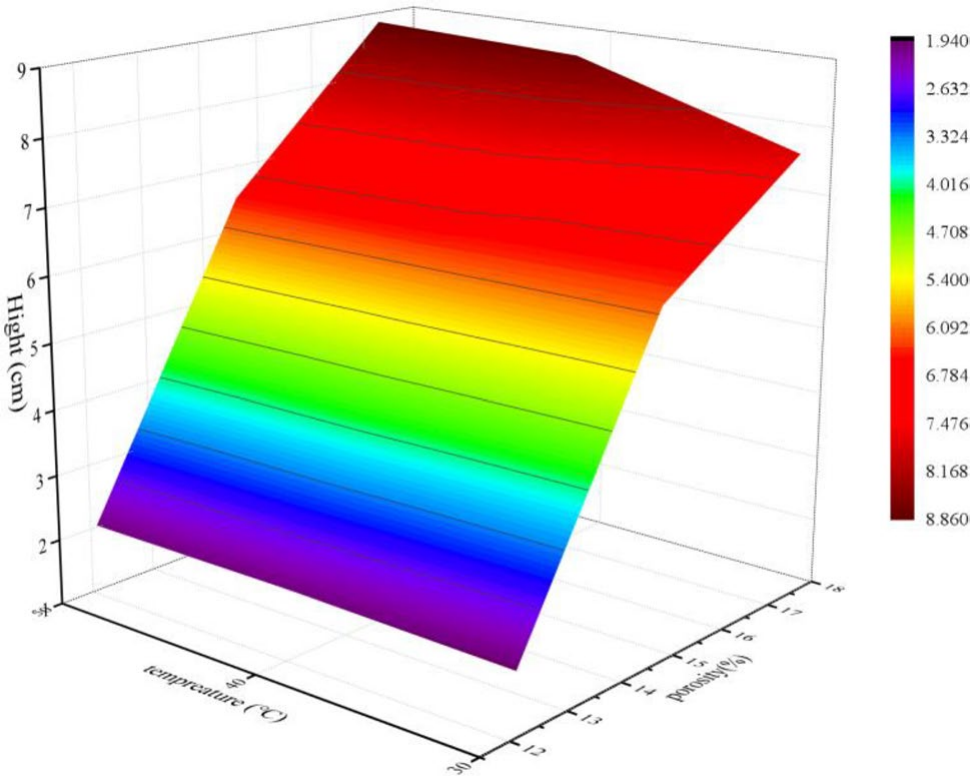
Coupling effect of porosity and temperature on capillary rise height.

Figure 9 presents the coupling effect of temperature and porosity on the final height of liquid capillary rise; the height is positively correlated with temperature and porosity. From this perspective, the liquid wetting ores is facilitated by high porosity and temperature. And in the stope leaching with large buried depth, the underground high-temperature environment is a promoting factor for the capillary penetration. Therefore, the liquid permeates the ore more rapidly during stope leaching in high geothermal areas, which can be used to identify the conditions for the preferred application of stope leaching. However, based on a sensitivity analysis, the rising height is more sensitive to porosity than to temperature. The effective connection between pores is a fundamental internal factor that affects capillary rise. In contrast, temperature is an external condition that can only slightly change the pore size or the flow characteristics. The increase in temperature changes the media characteristics and fluid properties, such as porosity, density, viscosity, and surface tension. It mainly affects the fluid fluidity, and the effect of temperature increase on the fluid fluidity is limited. The increase in porosity changes the structure of flow channel, such as pore diameter, pore length, pore throat, and pore tortuosity. The influence of the medium structure changes on the capillary penetration is far greater than the limited fluidity changes. Therefore, to ensure enhanced capillary permeability, the effective connectivity between pores need to be improved. During stope leaching, the effective porosity inside the rocks can be improved by increasing the number of secondary pores, which can be achieved through blasting vibration. However, excessive blasting vibration adversely affects mining safety and increases costs. To identify the appropriate extent of blasting vibration, comprehensive research on stope safety, mining cost, and leaching efficiency is needed.

## Conclusions

In this study, the liquid distribution characteristics of capillary rise inside particles were analysed using non-invasive magnetic resonance imaging technology, which is an effective technique. The liquid capillary rise process can be intuitively represented, and the pore water distribution characteristics can be quantitatively characterised.

The liquid capillary rise is significantly affected by the porosity. With an increase in porosity, the effective connectivity of pores and the capillary rise rate are substantially enhanced, and the liquid distribution profile changes. With an increase in temperature, the intra-particle liquid capillary rise increases slightly, whereas the liquid distribution profile remains unchanged. Therefore, to improve the efficiency of industrial stope leaching, the number of internal pores and cracks need to be increased cost-effectively. This can be achieved through enhanced blasting vibration during the caving process of mining to increase the number of secondary micro-cracks in the ores. Since the temperature range of industrial stope leaching is below the threshold, the exothermic leaching reaction and an increase in the ambient temperature facilitate the liquid infiltration of ores.

The limitation of exploring the liquid distribution inside particles using low-intensity NMR imaging equipment is that the entire sample is detected as a slice, and that the horizontal liquid distribution is not displayed completely. In subsequent studies, higher-intensity MRI equipment can be used to obtain the liquid distribution of multiple slices accurately. This article focuses on the intra-particle liquid capillary rise. The of liquid after pores saturation, which represents solute transfer between intra-particle liquid and active flow paths between the particles, is an important direction of follow-up research. This paper focuses on the liquid permeating ore, and the liquid drainage of ore remains to be further studied.
